# Supplementary material for: Measurement error associated with gait cycle selection in treadmill running at various speeds
Source: PeerJ. 2023 Mar 17;11:e14921. doi: 10.7717/peerj.14921 (PMC10026719; doi:10.7717/peerj.14921)
Supplement: Supplemental Information 1 — Figures demonstrate a comparison of errors across the various running speeds examined. Darker points and solid lines in figures equate to the mean ± standard deviation. Horizontal lines within boxes equate to the median value, boxes indicate the 25th to 75th percentile, and dashed whiskers indicate the range. Shaded violins are included to illustrate the distribution of values. [file peerj-11-14921-s001.pdf]

Measurement error associated with gait cycle selection in treadmill running at various speeds

Aaron S. Fox, Jason Bonacci, John Warmenhoven, Meghan F. Keast

Supplemental File 1

Figure S1: Absolute error in peak kinematic variables (i.e. zero-dimensional [0D]) when running at 2.5 m·s-1 (blue), 3.5 m·s-1 (yellow) and 4.5 m·s-1 (red) using a subset of gait cycles versus all gait cycles from the 30-second treadmill bout.

Figure S2: Peak absolute error in kinematic variables across the gait cycle (i.e. one-dimensional [1D]) when running at 2.5 m·s-1 (blue), 3.5 m·s-1 (yellow) and 4.5 m·s-1 (red) using a subset of gait cycles versus all gait cycles from the 30-second treadmill bout.

Figure S3: Absolute error in peak kinematic variables (i.e. zero-dimensional [0D]) when running at 2.5 m·s-1 (blue), 3.5 m·s-1 (yellow) and 4.5 m·s-1 (red) using two comparative subsets of gait cycles from the 30-second treadmill bout.

Figure S4: Peak absolute error in kinematic variables across the gait cycle (i.e. one-dimensional [1D]) when running at 2.5 m·s-1 (blue), 3.5 m·s-1 (yellow) and 4.5 m·s-1 (red) using two comparative subsets of gait cycles from the 30-second treadmill bout.

Darker points and solid lines in figures equate to the mean ± standard deviation. Horizontal lines within boxes equate to the median value, boxes indicate the 25th to 75th percentile, and dashed whiskers indicate the range. Shaded violins are included to illustrate the distribution of values.

Abbreviations: FLEX – flexion; EXT – extension; ADD – adduction; IR – internal rotation; ER – external rotation; DF – dorsiflexion; PF – plantarflexion.

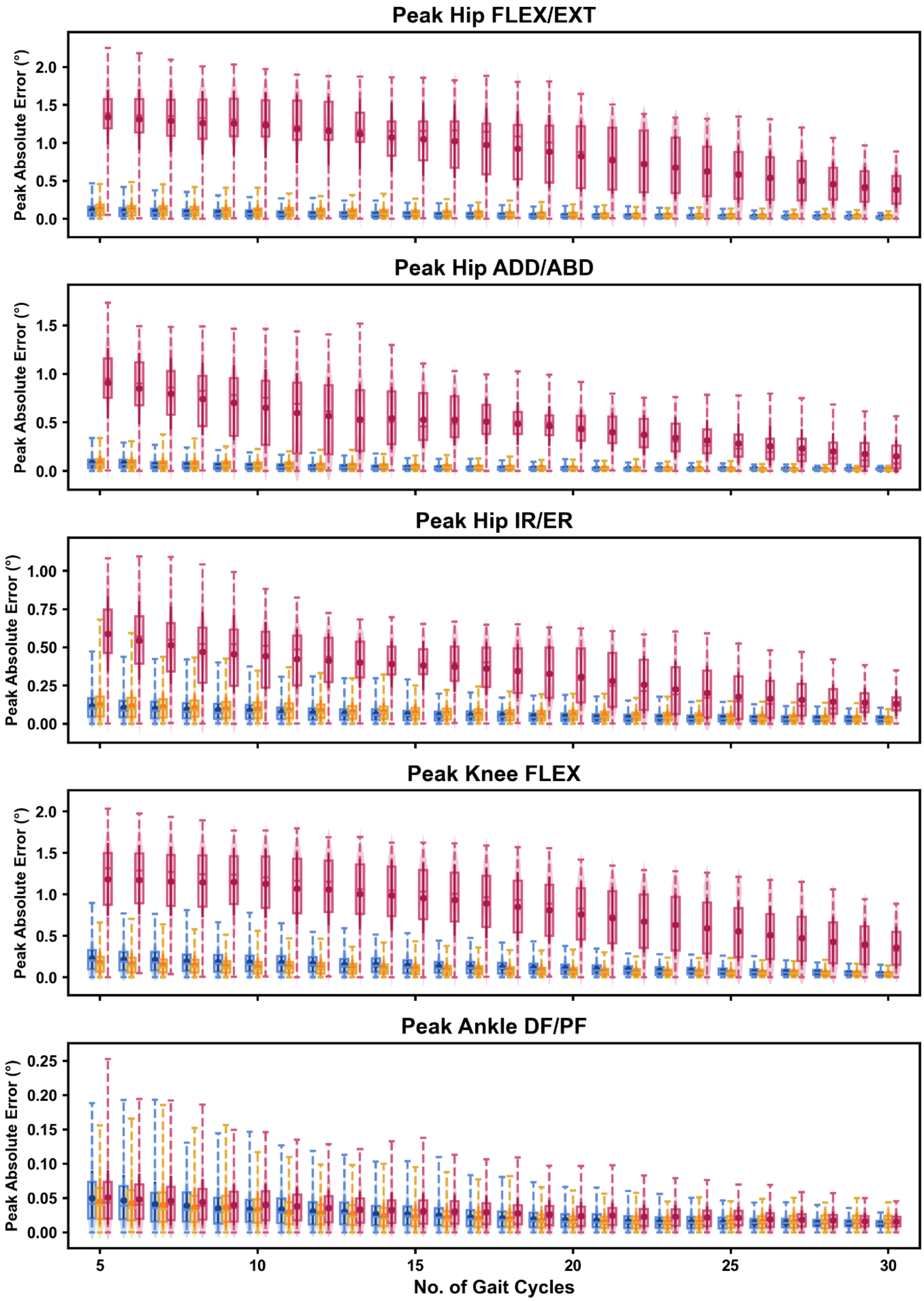

Figure S1: Absolute error in peak kinematic variables (i.e. zero-dimensional [0D]) when running at 2.5 m·s^{-1} (blue), 3.5 m·s^{-1} (yellow) and 4.5 m·s^{-1} (red) using a subset of gait cycles versus all gait cycles from the 30-second treadmill bout.

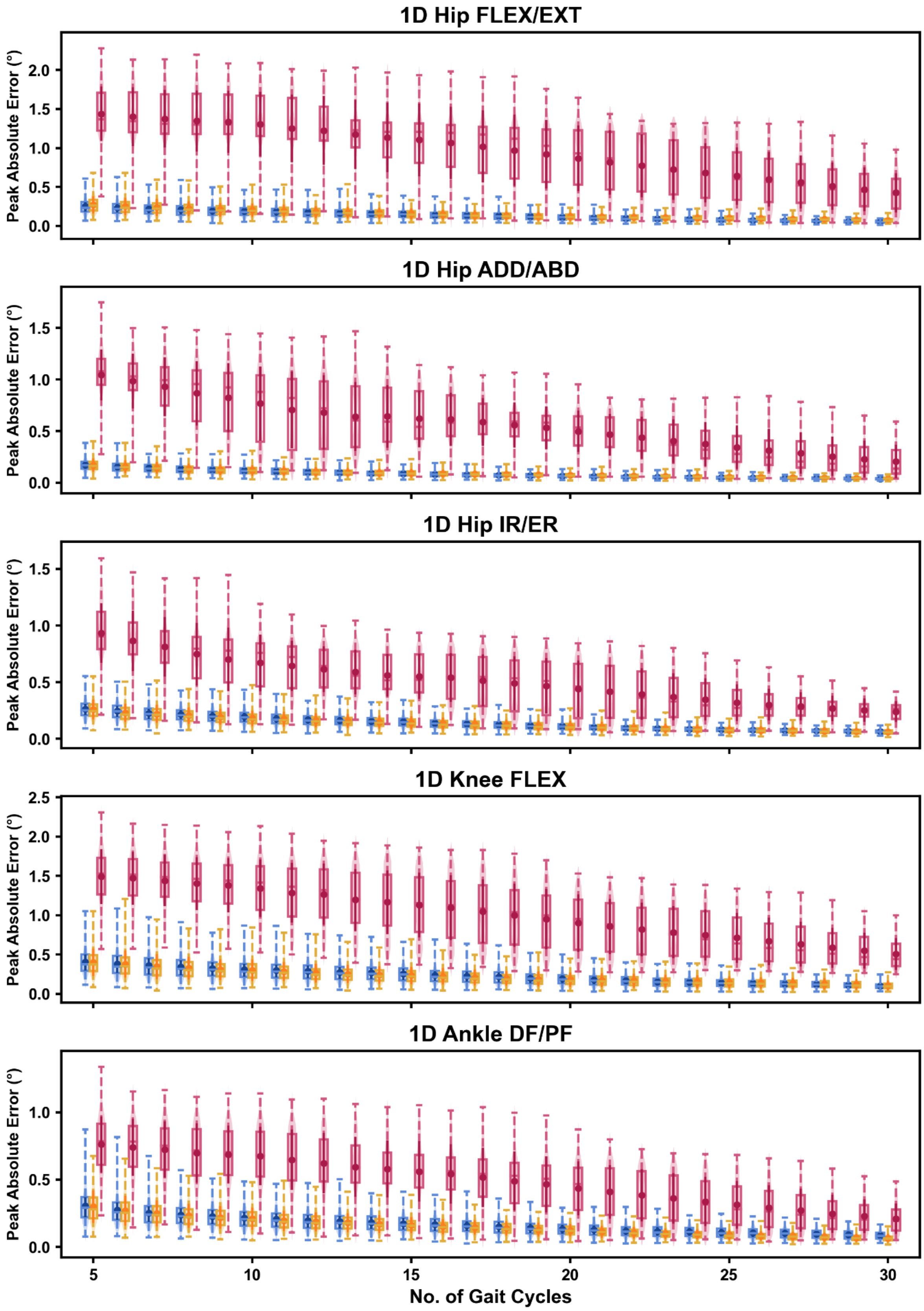

Figure S2: Peak absolute error in kinematic variables across the gait cycle (i.e. one-dimensional [1D]) when running at 2.5 m·s^{-1} (blue), 3.5 m·s^{-1} (yellow) and 4.5 m·s^{-1} (red) using a subset of gait cycles versus all gait cycles from the 30-second treadmill bout.

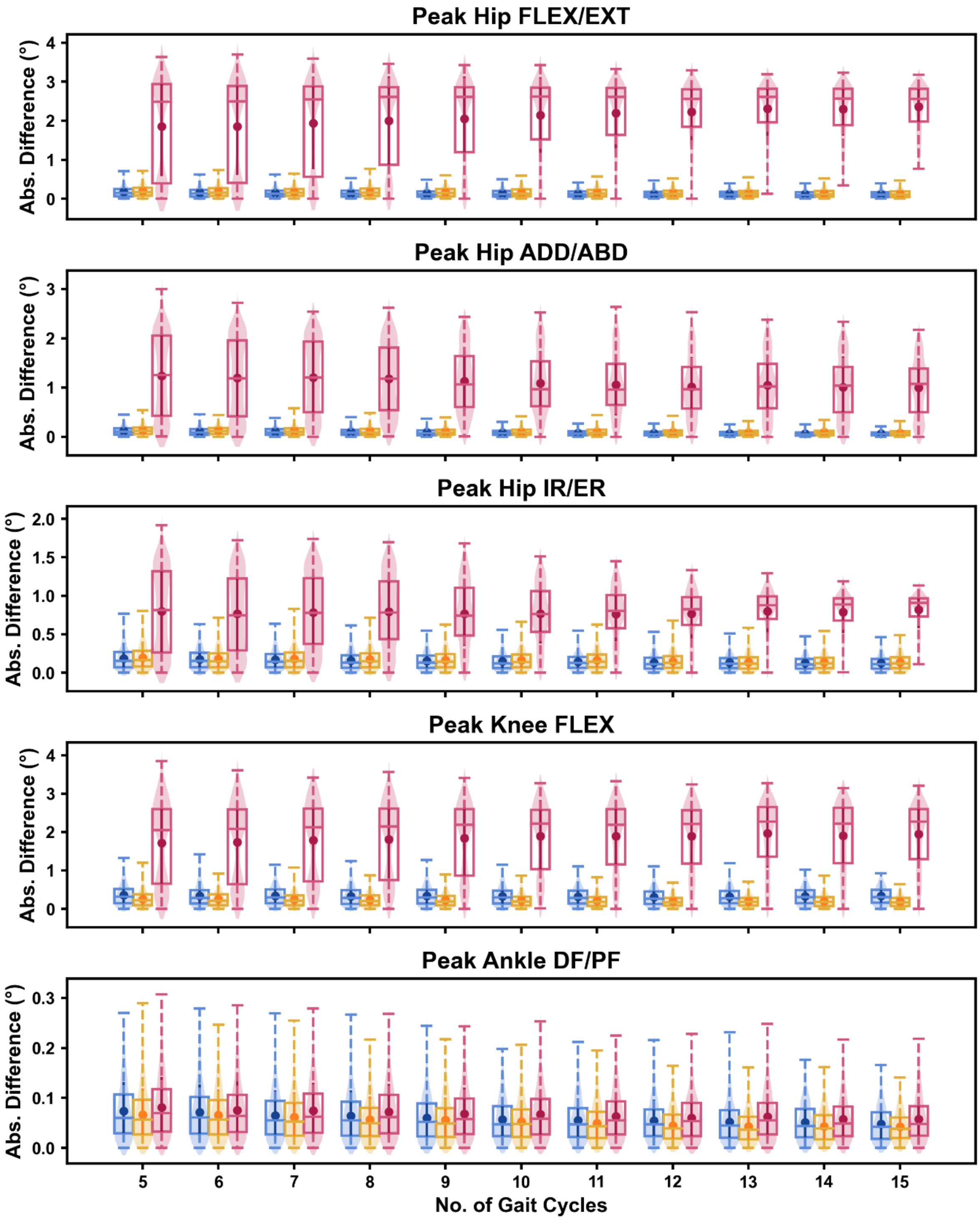

Figure S3: Absolute error in peak kinematic variables (i.e. zero-dimensional [0D]) when running at 2.5 m·s^{-1} (blue), 3.5 m·s^{-1} (yellow) and 4.5 m·s^{-1} (red) using two comparative subsets of gait cycles from the 30-second treadmill bout.

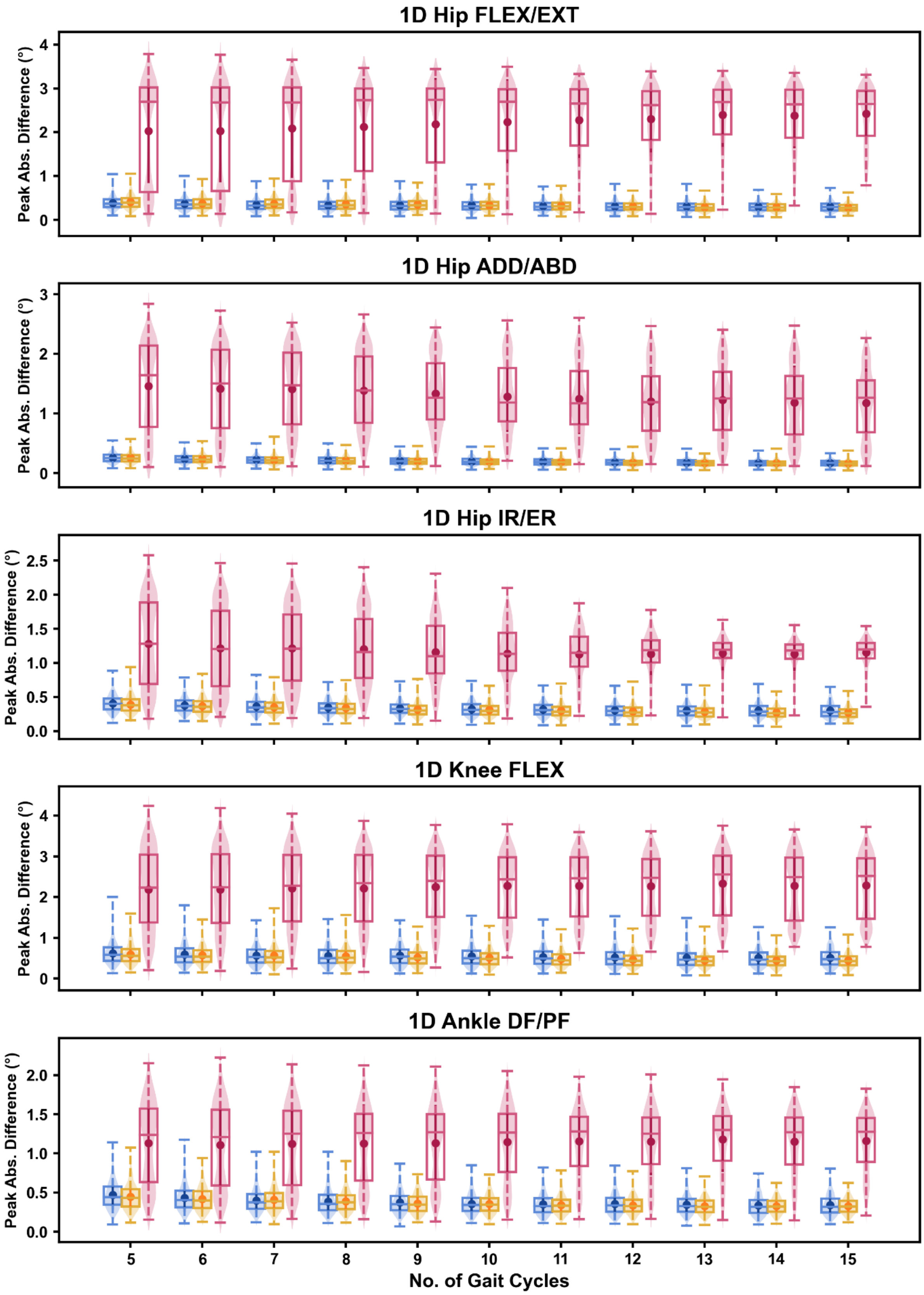

Figure S4: Peak absolute error in kinematic variables across the gait cycle (i.e. one-dimensional [1D]) when running at 2.5 m·s^{-1} (blue), 3.5 m·s^{-1} (yellow) and 4.5 m·s^{-1} (red) using two comparative subsets of gait cycles from the 30-second treadmill bout.
